# Association of rs2241766 and rs1501299 polymorphisms in the adiponectin gene with metabolic syndrome

**DOI:** 10.1002/iid3.70025

**Published:** 2024-09-18

**Authors:** Yinghua Tang, Lianli Yin, Faquan Lin

**Affiliations:** ^1^ Department of Clinical Laboratory The First Affiliated Hospital of Guangxi Medical University, Key Laboratory of Clinical Laboratory Medicine of Guangxi Department of Education Nanning Guangxi China; ^2^ Department of Clinical Laboratory The First Affiliated Hospital of Guangxi University of Chinese Medicine Nanning Guangxi China; ^3^ Department of Clinical Laboratory The People's Hospital of Guangxi Zhuang Autonomous Region, Guangxi Academy of Medical Sciences Nanning Guangxi China

**Keywords:** adiponectin, association, metabolic syndrome, polymorphisms

## Abstract

**Objective:**

To investigate the influence of adiponectin (APN) rs2241766 and rs1501299 polymorphisms on adiponectin levels and their association with metabolic syndrome (MetS).

**Methods:**

Analyzed two polymorphisms (rs2241766 and rs1501299) of the adiponectin gene (ADIPOQ) in 210 MetS patients and 102 control patients using the polymerase chain reaction‐restriction fragment length polymorphism method and DNA sequencing technology.

**Results:**

The genotypes of the rs2241766 T/G and rs1501299 G/T polymorphism were significantly associated with serum APN levels in MetS patients. The ADIPOQ polymorphisms were associated with a risk of MetS when compared with that in healthy controls. TG and GG genotypes of rs2241766 were associated with a significantly elevated risk of MetS as compared with the TT genotype (OR = 1.32 and OR = 2.53). Subjects with the G allele appeared to have higher susceptibility to MetS than those with the T allele (OR = 2.21). In common with the findings for rs2241766, the rs1501299 GT and TT genotypes were associated with a significantly increased risk of MetS as compared with the GG genotype (OR = 1.51 and OR = 2.24). The susceptibility to MetS appeared to be higher in subjects with the T allele than in those with the G allele (OR = 1.88).

**Conclusions:**

The occurrence of MetS may be associated with genetic variations at the rs2241766 and rs1501299 loci, especially in individuals with T to G mutations (rs2241766) and G to T mutations (rs1501299). These mutations may lead to decreased APN levels and a higher risk of developing MetS.

## Graphical abstract

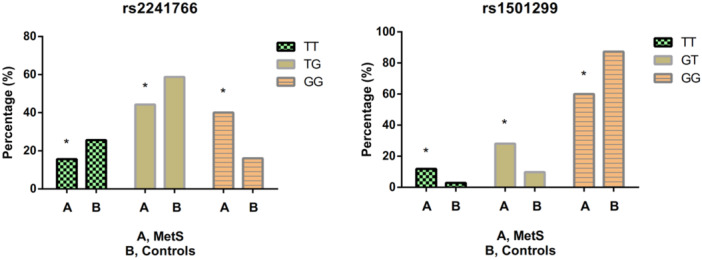

## 1 INTRODUCTION

Metabolic syndrome (MetS), which refers to a series of pathological and physiological changes caused by abnormal accumulation of various metabolic components in the body, is a cluster of complex metabolic disorders. MetS is associated with a 2–3 fold increased risk of heart disease.^1^ With improvements in people's living standards and lifestyle changes, the prevalence of MetS has continued to increase year on year.^2^ As a result, MetS has become a serious public health problem, which has attracted much research attention. Adiponectin (APN), a protein specifically secreted by adipose tissue, is associated with increased insulin sensitivity. APN also exhibits anti‐inflammatory, antiatherosclerotic, and antiplatelet aggregation properties. Autocrine, paracrine, and endocrine forms of APN are thought to influence insulin resistance, glucose conversion, lipid metabolism, vascular endothelial function, and inflammatory responses.^3^ Some scholars have proposed that the occurrence of Mets is closely related to APN levels and that the serum APN levels of Mets patients are significantly lower than those of healthy individuals.^4^ Therefore, some researchers believe that APN serves as a biological marker of MetS and that it plays an important role in the occurrence and development of MerS.^5^

In humans, the APN gene is located on chromosome 3q27, which is rich in polymorphisms and is a susceptibility gene region for type 2 diabetes mellitus, MetS, and coronary heart disease.^6^ Ethnic and regional differences in APN polymorphisms have resulted in inconsistent and even conflicting results for populations of the same race and different regions in studies conducted.^7^ The aim of the present study was to investigate the associations between two single nucleotide polymorphisms (SNPs), rs2241766 and rs1501299 of the APN gene ADIPOQ, and their association with APN levels and MetS. The resulting epidemiological and genetic data can aid understanding of the possible mechanism of the role of rs2241766 and rs1501299 in MetS patients.^8^

## 2 MATERIAL AND METHODS

### 2.1 Study population

From June 2019 to December 2021, 210 patients (females, *n* = 114; males, *n* = 96) with MetS aged 34–66 years (median age, 42.3 years), and 102 volunteer blood donors (control group) aged 28–61 years (median age, 39.2 years) were enrolled in this study. All the patients were diagnosed with MetS according to the 2016 Chinese guidelines for the management of dyslipidemia in adults. Based on these guidelines, patients with central obesity (waist circumference ≥90 cm for men and ≥85 cm for women), in addition to any two of the following parameters were included: elevated plasma triglycerides (TG) (≥1.70 mmol/L), low plasma high‐density lipoprotein cholesterol (HDL‐C) (<1.00 mmol/L), systolic blood pressure (SBP) ≥ 130 or diastolic blood pressure (DBP) ≥ 85 mmHg, fasting plasma glucose (FPG) ≥ 6.10 mmol/L, or diagnosed with type 2 diabetes mellitus. The healthy controls did not meet any of the criteria for MetS.

Blood samples were obtained from all the participants at the time of diagnosis. The clinical characteristics of the patients (age, sex, therapeutic interventions, etc.) were obtained from medical records. The weight, height, and visceral adiposity of the patients were also recorded. This research was approved by the Medical Ethics Committee of the First Affiliated Hospital of Guangxi University of Chinese Medicine (No: AF/SC‐08/04.0). All the procedures performed in studies involving human participants were in accordance with the ethical standards of the institutional and/or national research committee and with the 1964 Helsinki Declaration and its later amendments or comparable ethical standards. All the participants in the study provided written informed consent.

### 2.2 Measurements of biochemical parameters and genotyping

A venous blood sample (5 ml) was collected from each subject and placed in a dry ethylene diamine tetraacetic acid (EDTA) anticoagulant tube. Serum from the patients and healthy controls was separated from the cellular fraction by centrifugation at 2500 rpm. (1,500 × *g*) for 10 min and frozen at −20°C for the determination of APN. The blood in EDTA anticoagulant tubes was well mixed and used for DNA extraction.

APN levels were measured using an enzyme‐linked immuno sorbent assay kit (Tiangen Biotech, Beijing, China) and processed according to the manufacturer's instructions. Genomic DNA was isolated from whole blood samples using a TIANamp genomic DNA kit (Tiangen Biotech,) and processed according to the manufacturer's instructions. The DNA concentration was determined spectrophotometrically. Genotyping of APN was determined by the polymerase chain reaction (PCR) restriction fragment length polymorphism method. The PCR assay was performed using a Gene AmpPCR ABI 9700 system (ABI Applied Biosystems, USA). The APN genomic sequence (NT_005612.16) was obtained from the National Center for Biotechnology Information. For the APN rs2241766 and rs1501299 polymorphisms, the forward primer was 5′‐ CTTGGTGAGGAAAGGAGAC‐3′, and the reverse primer was 5′‐ GAGGAATCAGAATATGAATG‐3′. The final PCR mixture (50 μl) contained DNA (2.0 μl), PCR mixed reaction liquid (8.0 μl), 1.0 μl of each primer, Taq DNA polymerase (2.5 U/μl), and water. The thermocycler program consisted of an initial step of 94°C for 15 min, followed by 35 cycles of 94°C for 1 min, 55°C for 45 s, 72° C for 1 min, and a final extension step of 72°C for 5 min.

The enzymatic reaction system consisted of an amplified product (20.0 μl), restriction enzyme (1.0 μl) buffer (3.0 μl), and water (6.0 μl). The amplified DNA fragments were verified on a 2% agarose gel after incubation for 1 h at 37°C.

Each study participant was classified as having one of three possible genotypes. To validate the accuracy of the genotyping results, 70 random samples were repeatedly tested. There was 100% concordance between the results of the two tests. In addition, direct sequencing of 30 samples revealed 100% concordance with the genotyping results of the present study.

### 2.3 Statistical analysis

The genotype and allele frequencies of ADIPOQ in two groups were compared using the *χ*2 test. To assess the relative risk conferred by a particular allele and genotype, odds ratios (ORs) and their 95% confidence intervals (CIs) were calculated by binary logistic regression and adjusted for age. Demographic and clinical data among the groups were compared using a χ2 test and an analysis of variance. To compare the observed genotype frequencies among the subjects with expected genotype frequencies, the Hardy–Weinberg equilibrium was tested using the goodness of fit χ2 test. Differences in serum APN levels among the MetS patients and differences in serum APN levels between the MetS patients and controls were examined using a one‐way analysis of variance, and if significant, pairwise comparisons were performed. To elucidate the interaction between the factors studied (genotype and serum APN levels), the data were analyzed by a two‐way analysis of variance. All statistical analyses were performed using SPSS 21.0 (International Business Machines Corporation, USA).

## 3 RESULTS

### 3.1 Clinical characteristics of the subjects

The clinical characteristics of the two groups are shown in Table 1. There were significant differences in the height, weight, hip circumference, waist circumference, SBP, DBP, FPG, body mass index, TG, HDL‐C, low‐density lipoprotein cholesterol, and APN levels of the subjects in the different groups. There were no significant differences in the ages and heights of the participants, suggesting that the subjects were adequately matched based on these variables.

**Table 1:** Clinical characteristics of the study participants.

Indicator	MetS group (*n* = 210)	Control group (*n* = 102)	*p* value
Sex (F/M)	114/96	62/40	0.750
Age	42.3 ± 13.2	38.2 ± 13.8	0.185
Height (cm)	165.2 ± 12.9	166.8 ± 13.1	0.274
Weight (kg)	64.4 ± 6.3	61.2 ± 6.9	<0.001
Hip circumference (cm)	104.1 ± 11.2	93.5 ± 8.6	<0.001
Waist circumference (cm)	90.2 ± 7.1	83.3 ± 6.4	<0.001
SBP (mm Hg)	131.5 ± 19.2	108.6 ± 14.9	<0.001
DBP (mm Hg)	84.2 ± 12.1	74.1 ± 8.9	<0.001
FPG (mmol/L)	5.68 ± 2.02	4.66 ± 0.81	<0.001
BMI (kg/m^2^)	26.43 ± 2.58	22.12 ± 2.39	<0.001
TG (mmol/L)	2.89 ± 0.66	1.21 ± 0.52	<0.001
TC (mmol/L)	5.54 ± 1.76	4.55 ± 1.25	<0.001
LDL‐C (mmol/L)	3.52 ± 1.76	1.77 ± 0.95	<0.001
HDL‐C (mmol/L)	1.31 ± 0.41	1.68 ± 0.56	<0.001
APN (mg/L)	15.8 ± 4.2	26.8 ± 6.6	<0.001

APN, adiponectin; BMI, body mass index; DBP, diastolic blood pressure; FPG, fasting blood glucose; HDL‐C, high‐density lipoprotein cholesterol; LDL‐C, low‐density lipoprotein cholesterol; MetS, metabolic syndrome; SBP, systolic blood pressure; TC, total cholesterol; TG, triglycerides.

### 3.2 ADIPOQ genotypes and allele frequencies in the study groups

The genotype and allele frequencies of the ADIPOQ polymorphisms in the MetS patients and controls are summarized in Table 2 and Table 3. The genotype distributions of the rs2241766 T/G polymorphism and rs1501299 G/T polymorphism were consistent with Hardy‐Weinberg. Significant differences were observed in the distribution of the GG, TG, and TT genotypes at the rs2241766 locus between patients with MetS and the control group. Specifically, the frequencies of the GG, TG, and TT genotypes were significantly higher in MetS patients compared to the control group. Additionally, the frequencies of the G and T alleles were also significantly higher in the MetS patient group compared to the control group. Similarly, for the rs1501299 locus, there was a significant difference in the overall distribution of the GG, GT, and TT genotypes between MetS patients and the control group (*p* < .05). The frequencies of the GG, GT, and TT genotypes, as well as the G and T alleles, were also significantly higher in MetS patients compared to the control group (*p* < .05). The TG and GG genotypes of rs2241766 were associated with a increased risk of MetS as compared with the TT genotype (OR = 1.32, 95% CI 0.67–2.25; OR = 2.53, 95% CI 1.72–7.40 vs. OR = 1.00 ref). The G allele was associated with a significantly increased risk of MetS as compared with the T allele. Under the dominant model, genotype TG + GG appeared to be associated with an elevated risk of MetS when compared with the TT genotype (OR = 2.31, 95% CI 1.29–6.17). In common with rs2241766, the GT and TT genotypes of rs1501299 were associated with an elevated risk of MetS as compared with the GG genotype (OR = 1.51, 95% CI 0.84–2.50; OR = 2.24, 95% CI 1.35–5.92 vs. OR = 1.00 ref). The T allele was associated with a significantly increased risk of MetS as compared with the G allele. Under the dominant model, the genotype GT + TT appeared to be associated with an increased risk of MetS when compared with the GG genotype (OR = 1.78, 95% CI 1.22–4.81).

**Table 2:** Results of rs2241766 and rs1501299 gene testing in MetS patients and controls.

groups	*n*	SNP	genotypes			HW‐*p* value	Allele genes		SNP	genotypes			HW‐*p* value	Allele genes	
			TT	TG	GG		T	G		TT	GT	GG		T	G
MetS	210	rs2241766	33 (15.6)*	93 (44.3)*	84 (40.1)*	0.087	159 (37.9)*	261 (62.1)*	rs1501299	25 (11.9)*	59 (28.1)*	126 (60.0)*	0.089	109 (26.0)*	311 (74.0)*
Controls	102		26 (25.6)	60 (58.8)	16 (16.1)	0.081	112 (54.9)	92 (45.1)		3 (2.9)	10 (9.8)	89 (87.3)	0.073	16 (7.8)	188 (92.2)

* Compared with the controls, *p* < .05; MetS, metabolic syndrome; HW, Hardy‐Weinberg; SNP, single nucleotide polymorphisms.

**Table 3:** Genotype and allele frequencies of the two SNPs in the ADIPOQ in MetS patients and controls.

SNP	Polymorphisms	MetS patients	Controls	OR (95% CI)	*p* value
		*n* = 210 (%)	*n* = 102 (%)		
rs2241766	TT	33 (15.6)	26 (25.6)	1.00 ref	
	TG	93 (44.3)	60 (58.8)	1.32 (0.67–2.25)	0.219
	GG	84 (40.1)	16 (16.1)	2.53 (1.72–7.40)	0.000
	TG + GG	177 (84.3)	76 (74.5)	2.31 (1.29–6.17)	0.025
	T allele	159 (37.9)	112 (54.9)	1.00 ref	
	G allele	261 (62.1)	92 (45.1)	2.21 (1.19–3.03)	0.038
rs1501299	GG	126 (60.0)	89 (87.3)	1.00 ref	
	GT	59 (28.1)	10 (9.8)	1.51 (0.84–2.50)	0.296
	TT	25 (11.9)	3 (2.9)	2.24 (1.35–5.92)	0.016
	GT + TT	84 (40.0)	13 (12.7)	1.78 (1.22–4.81)	0.024
	G allele	311 (74.0)	188 (92.2)	1.00 ref	
	T allele	109 (26.0)	16 (7.8)	1.88 (1.26–3.98)	0.044

MetS, metabolic syndrome; 95% CI, 95% confidence interval; OR, odds ratio; SNP, single nucleotide polymorphisms.

### 3.3 Association between ADIPOQ polymorphisms and serum APN levels in MetS patients

As shown in Table 4, serum APN levels were associated with ADIPOQ polymorphisms in MetS patients, with a significant association observed between the genotypes of the rs2241766 T/G and rs1501299 G/T polymorphisms and serum APN levels. Serum APN levels were significantly lower in individuals with a homozygous GG genotype (14.5 ± 4.3 mg/mL) or heterozygous TG genotype (15.9 ± 4.2 mg/mL) than a homozygous TT genotype (18.4 ± 4.7 mg/mL) of the rs2241766 polymorphism (*p* < .05). Similarly, serum APN levels were significantly lower in individuals with a homozygous TT genotype (13.7 ± 5.4 mg/mL) or a heterozygous GT genotype (14.1 ± 5.3 mg/mL) than a homozygous GG genotype (15.9 ± 4.9 pg/mL) of the rs1501299 polymorphism (*p* < .05).

**Table 4:** The association between ADIPOQ polymorphisms and serum APN concentrations in MetS patients.

SNP	Polymorphisms	MetS patients	APN (mg/mL)	*p* value
		*n* = 210 (%)	*n* = 210	
rs2241766	TT	33 (15.6)	18.4 ± 4.7	
	TG	93 (44.3)	15.9 ± 4.2	<0.001
	GG	84 (40.1)	14.5 ± 4.3	<0.001
	TG + GG	177 (84.3)	15.1 ± 5.2	<0.001
rs1501299	GG	126 (60.0)	15.9 ± 4.9	
	GT	59 (28.1)	14.1 ± 5.3	<0.001
	TT	25 (11.9)	13.7 ± 5.4	<0.001
	GT + TT	84 (40.0)	13.9 ± 5.5	<0.001

ADIPOQ, adiponectin gene; MetS, metabolic syndrome; APN, adiponectin; SNP, single nucleotide polymorphisms.

## 4 DISCUSSION

MetS is a cluster of complex metabolic disorders, resulting from a series of pathological and physiological changes caused by abnormal accumulation of various metabolic components in the body. Its prevalence among adults has increased from 25.3% to 35.8% in the United StatesIn recent years.^9^ Due to lifestyle changes in human, the prevalence of MetS is rising rapidly. MetS is a risk factor for cardiovascular disease (CVD) and is associated with a 2–3 fold increased risk of heart disease and death.^10^ Many patients with MetS eventually develop CVD and kidney disease, which are leading a large social and economic burden on many countries.^11^ As such, there is an urgent need to determine the potential risk factors for predicting which patients without clinical symptoms are likely to develop MetS.

Adipose tissue can secrete a wide range of biologically active cytokines, which termed adipokines. Besides being involved in fat metabolism, many adipokines have pro‐inflammatory or anti‐inflammatory functions.^12^, ^13^ With an increase in adiposity, the expression of pro‐inflammatory adipokines is enhanced, whereas that of anti‐inflammatory adipokines is reduced. Classical examples of pro‐inflammatory adipokines are APN.^14^ APN promotes metabolic functions and provides cardiovascular protection.^15^ Many of the protective actions of APN are attributed to its anti‐inflammatory properties. Ouichi and Walsh reported that APN inhibited nuclear factor κB (NF‐κB) activation in endothelial cells following treatment with pro‐inflammatory factors, such as TNF‐α.^16^ Wang et al. reported that APN appeared to protect the aorta from atherosclerotic injury by reducing inflammation and suggested that the molecular mechanism may involve inhibition of the expression of downstream components of NF‐κB.^17^

The APN gene, which is rich in polymorphisms, is located on human chromosome 3q27 in a susceptibility gene region for type 2 diabetes mellitus and MetS.^18^ The present study aimed to identify APN polymorphisms associated with MetS and to determine whether these APN polymorphisms were related to the occurrence of MetS in this population. The selection criteria for the two SNPs were that they were located in exon or intron regions and that single nucleotide changes in these SNPs resulted in an amino acid substitution that led to decreased synthesis and secretion of APN. In this study, serum APN levels of the MetS group were significantly reduced as compared with those of healthy controls. This finding is in agreement with that of previous studies,^19^, ^20^, ^21^ suggesting that low serum APN levels appear to be closely associated with the occurrence of MetS. Furthermore, it suggests that APN plays an important role in preventing the development and progression of MetS.

The rs2241766 and rs1501299 polymorphisms are common SNPs of ADIPOQ. The rs2241766 polymorphism is a T/G substitution in exon 2, and the rs1501299 polymorphism is a G/T substitution in intron 2. Although there is evidence for an association between rs2241766 and an elevated risk of MetS, the underlying molecular mechanism remains unclear.^22^ The rs2241766 polymorphism, which is located in exon 2 and results in a synonymous change (G15G), is relatively close to the exon‐intron boundary.^23^ Therefore, it may affect the splicing machinery. There is increasing evidence that even silent mutations in coding regions may modify RNA levels by affecting splicing and thus decreasing expression of the gene. In this regard, a bioinformatics analysis identified a consensus sequence recognized by a functional exonic splicing‐enhancer in which 87% of the sequence matched to a 4 bp sequence in the 3′ region of the rs2241766 polymorphism.^24^ Therefore, rs2241766 may influence the expression of the APN gene and be associated with MetS. In common with the rs2241766 polymorphism, little is known about the molecular mechanism of rs1501299. Although rs1501299 is located in an intronic region, with no apparent biological function, this SNP may affect the expression level of the ADIPOQ gene through some unknown mechanisms. Alternatively, there may be undiscovered SNPs in the ADIPOQ gene or other genes that have biological effects on insulin resistance.

This study found significant differences in the genotype distribution between patients with MetS and healthy controls at two single‐nucleotide polymorphism (SNP) sites, rs2241766 and rs1501299. For rs2241766, the frequencies of the GG, TG, and TT genotypes in MetS patients were significantly higher than in the control group. Similarly, the frequencies of the G and T alleles were also significantly higher in the MetS patient group compared to healthy controls. These results suggest that these genotypes and alleles may play a promoting role in the development of MetS. Similarly, significant statistical differences were also observed at the rs1501299 site, where the frequencies of the GG, GT, and TT genotypes were also higher in MetS patients than in the control group. Additionally, the frequencies of the G and T alleles at this site were significantly higher in MetS patients compared to controls. The findings at these two sites not only reveal a specific association between certain genotypes and MetS but also emphasize the potential role of the G and T alleles in the pathophysiological processes. The high frequency of these genotypes and alleles may increase the risk of MetS by affecting key metabolic pathways such as lipid metabolism, insulin signaling, or inflammatory responses. In addition, further analysis of the association of the rs2241766 SNP with serum APN levels revealed that serum APN levels were significantly lower in individuals with the G genotype. This finding suggested that individuals carrying the G genotype with low to medium APN levels were more likely to develop MetS and that this genotype was a risk factor for MetS. This finding is in accordance with that of previous studies,^22^ suggesting that rs2241766 is associated with susceptibility to MetS patients.

The literature on the association of the G/T mutation at the rs1501299 locus of APN intron 3 with MetS is inconsistent. Some studies showed that the G/T mutation at the rs1501299 locus was related to MetS,^25^ whereas another study found no such relationship.^26^ In this study, the distribution frequencies of GT and TT genotypes of rs1501299 in MetS patients were significantly lower than those in the healthy controls. In addition, the T allele was more common in the MetS patients than in the controls. Further analysis of the association of the rs1501299 SNP with serum APN levels revealed that APN levels were significantly lower in individuals with the T genotype, suggesting that individuals carrying the G genotype with a low to medium level of APN were more likely to develop MetS and that this genotype was a risk factor for MetS.

This study has several limitations. First, the relatively small number of study participants is one of them. Additionally, all samples in this study come from patients at a single tertiary unit, and all subjects belong to the same ethnicity, which necessitates further research to determine if there are racial differences.

## 5 CONCLUSION

The variation at the rs2241766 and rs1501299 locus of the adiponectin gene is significantly associated with an increased risk of MetS. Especially individuals with T to G mutations (rs2241766) and G to T mutations (rs1501299) may lead to a decrease in APN levels and are more prone to MetS, indicating that these mutations are risk factors for MetS.

## AUTHOR CONTRIBUTIONS

Conceived and designed the experiments: FQ Lin. Performed the experiments: YH Tang. Analyzed the data: LL Yin. Contributed reagents/materials/analysis tools: FQ Lin. Wrote the paper: YH Tang. Final approval of the version to be submitted: FQ Lin.

## FUNDING

This study was supported by Guangxi Natural Science Foundation Project (2023GXNSFAA026124), and the Health Commission of the Guangxi Zhuang Autonomous Region (Z‐A20220039).

## CONFLICTS OF INTEREST STATEMENT

The authors declare that they have no conflict of interest.

## Supporting information

Supporting information.

## Data Availability

Data will be made available on request.
